# Problematic internet use and aggression in Chinese middle school students: mediation effect of reality social connectedness

**DOI:** 10.3389/fpubh.2025.1587400

**Published:** 2025-07-15

**Authors:** Junzhe Ran, Jiaqi Xu, Dan Luo, Tao Li, Jiajun Xu

**Affiliations:** ^1^The Mental Health Center, West China Hospital, Sichuan University, Chengdu, China; ^2^Psychiatric Laboratory, West China Hospital, Sichuan University, Chengdu, China; ^3^Department of Neurobiology, Affiliated Mental Health Center & Hangzhou Seventh People’s Hospital, Zhejiang University School of Medicine, Hangzhou, China; ^4^NHC and CAMS Key Laboratory of Medical Neurobiology, MOE Frontier Science Center for Brain Science and Brain-Machine Integration, School of Brain Science and Brain Medicine, Zhejiang University, Hangzhou, China

**Keywords:** problematic internet use, aggression, reality social connectedness, mediation effect, epidemiological research

## Abstract

**Introduction:**

Problematic internet use (PIU) has become a prevalent concern worldwide and is associated with increased aggression. However, the underlying effect of PIU on aggression remains unclear. In this study, we aimed to investigate the potential influence of reality social connectedness (RSC) on the relationship between PIU and aggression.

**Methods:**

We used cross-sectional data from a large survey conducted among middle school students in four provinces of China between September 2022 and March 2023. PIU, RSC, and aggression were assessed using Young’s 20-item Internet Addiction Test (IAT-20), the modified Social Connectedness Scale-Revised (SCS-R), and the Buss–Perry Aggression Questionnaire (BPAQ), respectively.

**Results:**

We found that students who experienced PIU had significantly higher scores on the BPAQ, which reflects the aggression levels, compared to students without PIU. Specifically, all four dimensions of aggression—verbal aggression, physical aggression, hostility, and anger—were elevated in the PIU group. Additionally, RSC was significantly reduced among individuals with PIU. Notably, RSC significantly mediated the relationship between PIU and aggression, accounting for 18.89% of the total effect. Among the four dimensions of aggression, the mediating effect of RSC was strongest for hostility, followed by anger and physical aggression, with the weakest observed for verbal aggression.

**Discussion:**

RSC significantly mediated the relationship between PIU and aggression, suggesting that reduced RSC partially explains how PIU exacerbates aggression. This result highlights the importance of fostering RSC as a strategy to reduce aggression related to PIU.

## 1 Introduction

Problematic internet use (PIU), often referred to as internet addiction, has become a prevalent concern in modern society, particularly among adolescents. The extensive use of the internet for various activities, including social media, online gaming, and browsing, has been linked to various psychological and behavioral issues (1–3).

Previous studies have shown a potential link between PIU and aggression (4–8) across various locations and cultures, highlighting this phenomenon as a universal issue. Aggression, which includes behaviors such as physical aggression, verbal aggression, hostility, and anger (9), represents a significant social problem, particularly among adolescents. Adolescence is a period of rapid psychosomatic development and identity formation, influenced by unique sociocultural factors. During this period, adolescents often face emotional instability and are prone to impulsivity (10), which can exacerbate aggressive thoughts and behaviors (11), creating challenges for not only the individuals themselves but also for those around them. The relationship between PIU and aggression is complex and multifaceted, influenced by various social and psychological factors (4, 7, 12). Although bidirectional influences between PIU and aggression have been reported, recent longitudinal studies mainly focused on the negative impact of PIU and indicated that excessive internet use typically precedes increases in externalizing behaviors (13, 14). A typical Interaction of Person-Affect-Cognition-Execution (I-PACE) model also posits dysregulated internet use as an upstream factor (15). However, further mechanisms underlying PIU and aggression were unclear. Identifying potential mediators in the direction from PIU to aggression is crucial, as it may provide important insights for interventions and guide future research.

Social connectedness, defined as “the sense of belonging and subjective psychological bond that people feel in relation to individuals and groups of others (16),” is fundamental to human beings. Previous studies have shown its positive effect on life expectancy (17–20). Additionally, social connectedness has been linked to reduced depression (21, 22) and a lower risk of cognitive decline in older adults (23). Notably, one study reported a decrease in social connectedness among individuals with PIU (24). Furthermore, increased social connectedness has been shown to reduce aggression (25, 26).

With the widespread use of the internet, online communities have gradually evolved into virtual societies. Consequently, a sense of virtual social connectedness has emerged, which is distinct from reality social connectedness (RSC) (27, 28). However, the commonly used Social Connectedness Scale (SCS), originally developed before the widespread adoption of the internet (prior to 2000), does not account for this distinction (29). As a result, previous studies did not distinguish between reality and virtual social connectedness.

Given this gap, our study aimed to investigate the role of RSC in the relationship between PIU and aggression among Chinese middle school students. We hypothesized that RSC would mediate the relationship between PIU and aggression, and this mediating effect would vary across four subtypes of aggression (physical aggression, verbal aggression, hostility, and anger). Understanding this underlying mechanism may provide a new insight into interevent the aggression with PIU.

## 2 Methods

### 2.1 Participants

Data for this study were derived from a large cross-sectional study that employed a convenience sampling approach among middle school students in four provinces of China—Fujian, Jiangsu, Sichuan, and Xinjiang—representing the eastern, southern, western, and northern regions, respectively (30). Briefly, one or two middle schools were selected from each province following consultation with local education bureaus. All students aged 12 to 19 years whose parents and themselves signed informed consent were then asked to complete an anonymous online questionnaire.

The inclusion criteria for this study were as follows: (1) adolescents aged 12–19 years who were enrolled in school during the study, and (2) adolescents who provided clear responses to all questionnaire items. The exclusion criteria were: (1) participants who completed the questionnaire survey in less than 10 min, (2) participants who submitted multiple survey questionnaire responses, and (3) participants who responded, “completely untrue” or “untrue” to the final item assessing response validity: “To what extent does your survey reflect your actual situation?”

Ethical approval for this study was obtained from the Ethics Committee of West China Hospital, Sichuan University (No. 2019-907).

### 2.2 Measurements

#### 2.2.1 Sociodemographic information

Sociodemographic information was collected for all participants, including age (in years), sex (boys/girls), place of residence (city/town/rural), self-reported subjective family economic situation (very poor/relatively poor/average/economically well off/economically well off), whether they were from an only-child family (yes/no), and the parents’ education level (illiterate/primary school/middle school/high school/technical secondary schools/junior college/bachelor/master/doctor).

#### 2.2.2 Assessment of PIU, aggression, and RSC

PIU was assessed using Young’s 20-item Internet Addiction Test (IAT), a widely used self-assessment scale (31). Each item is rated on a 5-point Likert scale ranging from 1 = “never,” 2 = “seldom,” 3 = “sometimes,” 4 = “often,” to 5 = “very often.” All items were summed to create the IAT score, yielding a score range from 20 to 100. The higher the score on the scale, the stronger the state of PIU exhibited by the subject. In this study, we set the threshold for PIU based on previous research, defining it as the IAT score equal to or greater than 50 points (32). In this study, Cronbach’s *α* was 0.937.

RSC was measured by the SCS-Revised (SCS-R) with a little modification, which contains 20 6-point Likert items (29). Compared to the raw version of SCS-R, we added the prompt “in the real world” to the questionnaire and increased the font size and boldness of the text to ensure effective communication with participants. During the total score calculated, items 3, 6, 7, 9, 11, 13, 15, 17, 18, and 20 are reversed scored. The total score (range from 20 to 120) was calculated, as a higher score means that participants have higher RSC with others. In this study, Cronbach’s *α* was 0.902. An exploratory factor analysis (EFA) was conducted, yielding a Kaiser–Meyer–Olkin (KMO) measure of sampling adequacy of 0.951, indicating excellent suitability for factor analysis. The cumulative variance explained by the extracted factors was 64.3%.

Aggression was measured using the Buss and Perry Aggression Questionnaire (9) (BPAQ), developed by Buss and Perry in 1992, which includes 29 items covering the four domains—physical aggression, verbal aggression, anger, and hostility. Items were scored on a 5-point Likert scale from 1 = “very non-compliant” to 5 = “very compliant,” with higher scores implying more aggressive behaviors. Physical aggression was calculated from questions 2, 5, 8, 11, 13, 16 (reverse scored), 22, 25, and 29; verbal aggression was derived from questions 4, 6, 14, 21, and 27; anger was obtained from questions 1, 9 (reverse scored), 12, 18, 19, 23, and 28; and hostility was derived from questions 3, 7, 10, 15, 17, 20, 24, and 26. The total score of BPAQ can reflect the overall aggression level of the subjects. In this study, Cronbach’s *α* was 0.917.

#### 2.2.3 Data analysis

For categorical variables, we used the frequency and percentage to show the result. For continuous variables, we used the Anderson–Darling test to detect whether the normal distribution is satisfied or not. The continuous variables following the normal distribution, the mean, and standard deviation (mean ± SD) were reported; otherwise, median and interquartile (IQR) will be reported.

Partial correlations between variables were calculated, with age and gender included as control variables. To better understand the relationship between PIU and aggression, especially for exclude the potential confounding factors, we employed hierarchical line regression. First, univariate linear regression was used to find out the potential associated with aggression. Then those factors were taken into the hierarchical linear regression. In step 1, the PIU status was set as the independent variable, and the BPAQ total score was set as the dependent variable. In step 2, we introduced adjusted variables, including sex, age, place of residence, parents’ education level, and self-reported family economic situation. Categorical variables, such as gender, place of residence, parents’ education level, and self-reported family economic situation, were represented using dummy variables. Finally, RSC was added to the regression model. In the regression analysis, the 95% confidence intervals were included, and both the *R*^2^ and adjusted *R*^2^ were calculated.

To evaluate the role of RSC in relationship between RSC and aggression, mediation analysis was performed using the bruceR (33) package, which is based on the mediation package in R (34). Mediation analysis helped determine whether the relationship between independent variables and dependent variables is through an indirect way. The PIU status was set as the independent variable, and the BPAQ total score and its four dimensions—physical aggression, verbal aggression, anger, and hostility—were set as the dependent variables separately. The RSC was set as the moderator variable. During mediation analysis, age, gender, residence, family economy, and adjusting for sociodemographic factors based on the results obtained from the previous hierarchical analysis were set as the co-variables.

Visualization of the data was performed using the R package “ggpubr” (35). All data analysis were produced by the RStudio (Version 2023.06.2 + 561, ^©^2009–2019 RStudio, Inc., Boston, MA, USA) with R (version 4.3.1) (36).

## 3 Results

### 3.1 Sample characteristics

A total of 11,945 questionnaires were collected. After excluding 2,057 low-quality responses and 480 participants who did not meet the age criteria, 9,391 (4,556 boys and 4,835 girls) valid questionnaires remained. About 21.89% of middle school students were PIU participants according to the IAT test. Significant differences were observed between the PIU group and the health group regarding sex, only-child status, residence, family economy, and mother’s educational level (Table 1).

**Table 1:** Basic demographic characteristics of participant.

Variable	Health (*N* = 7,336)	PIU (*N* = 2,055)	*p*-value
Age	15 (13, 16)	15 (14, 16)	<0.001***
Gender			0.018**
Girls	3,825 (52.1%)	1,010 (49.1%)	
Boys	3,511 (47.9%)	1,045 (50.9%)	
Only child			0.046*
No	4,079 (55.6%)	1,091 (53.1%)	
Yes	3,257 (44.4%)	964 (46.9%)	
Residence			<0.001***
Town	974 (13.3%)	288 (14%)	
City	5,870 (80%)	1,546 (75.2%)	
Village	492 (6.7%)	221 (10.8%)	
Family economy			<0.001***
Average	5,442 (74.2%)	1,525 (74.2%)	
Very poor	76 (1%)	32 (1.6%)	
Quite poor	344 (4.7%)	153 (7.4%)	
Relatively affluent	1,327 (18.1%)	322 (15.7%)	
Very affluent	147 (2%)	23 (1.1%)	
Father education level			0.206
Primary or below	610 (8.3%)	195 (9.5%)	
Secondary education	3,895 (53.1%)	1,063 (51.7%)	
Tertiary education	2,831 (27.6%)	797 (38.8%)	
Mother education level			0.036*
Primary or below	855 (11.7%)	282 (13.7%)	
Secondary education	3,652 (49.8%)	988 (48.1%)	
Tertiary education	2,829 (38.6%)	785 (38.2%)	

*p < 0.05, **p < 0.01, and ***p < 0.001.

### 3.2 Correlation among aggression, PIU, and RSC

**Figure 1:**
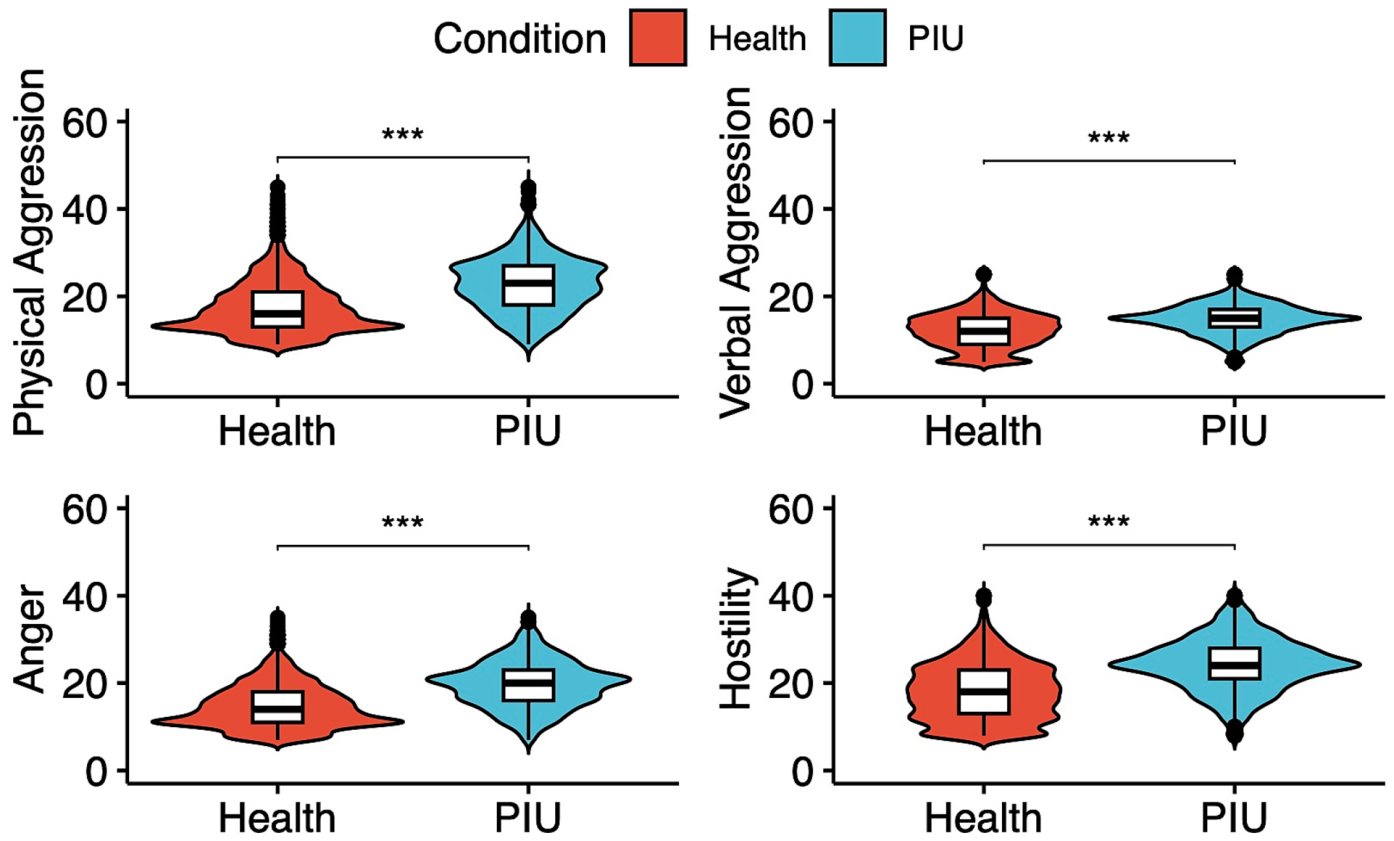
Four dimensions of aggression in PIU and healthy adolescents. ^*^p < 0.05, ^**^p < 0.01, and ^***^p < 0.001.

With regard to the health group, the total aggression scores of the PIU group were significantly higher [median (IQR) 61.0 (48.0 to 74.0) vs. 83.0 (70.0 to 91.0), *p* < 0.001]. Analysis of subsequent subscales indicated that the PIU participants showed significantly higher in four dimensions. Among PIU participants, the median (interquartile) scores for physical aggression, verbal aggression, anger, and hostility were 23.0 (18.0 to 27.0), 15.0 (13.0 to 17.0), 20.0 (16.0 to 23.0), and 24.0 (21.0 to 28.0), respectively. In contrast, among healthy individuals, the corresponding median (interquartile) scores were 16.0 (13.0 to 21.0), 12.0 (9.0 to 15.0), 14.0 (11.0 to 18.0), and 18.0 (13.0 to 23.0), respectively (Figure 1).

**Figure 2:**
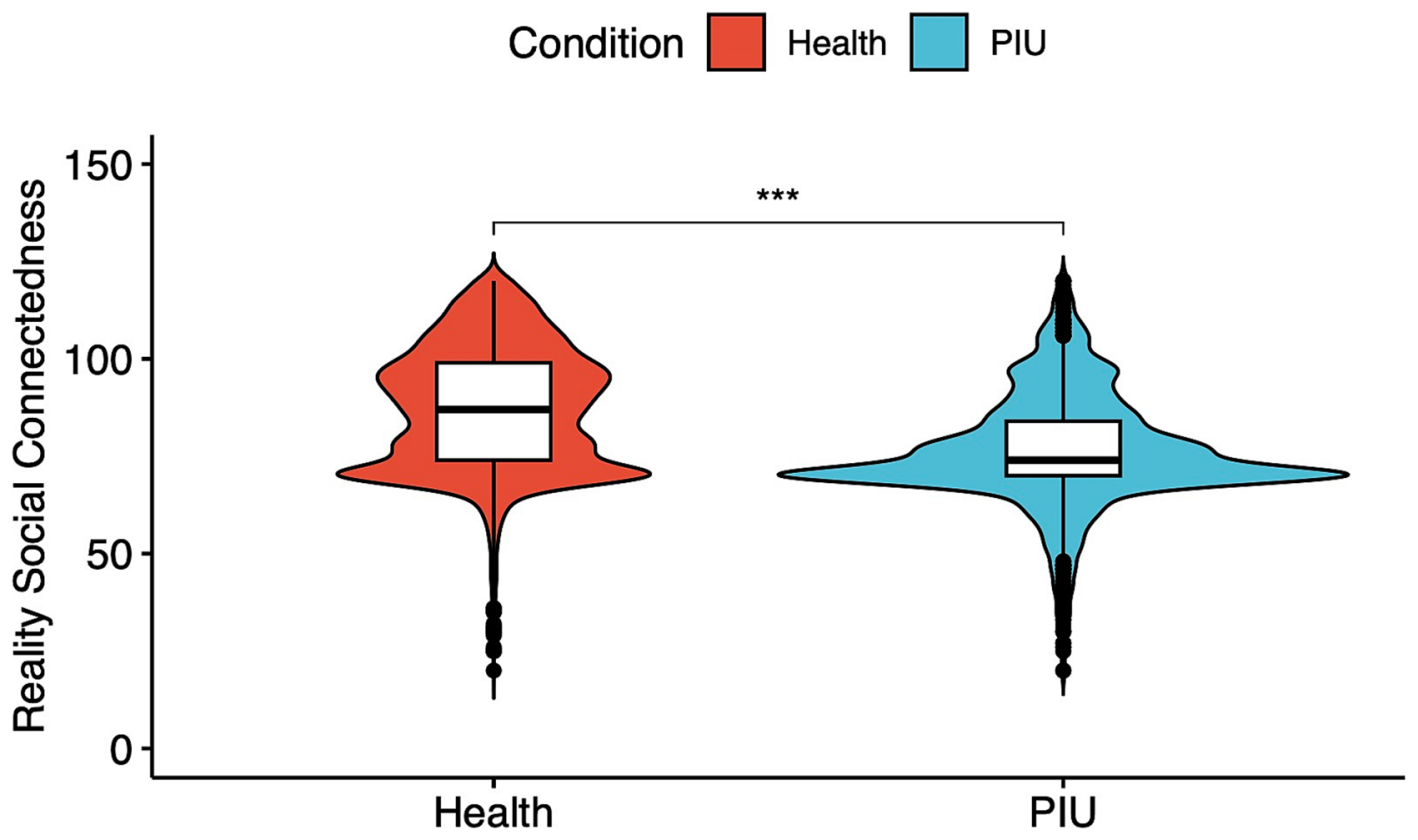
The RSC in PIU or healthy adolescents. ^*^p < 0.05, ^**^p < 0.01, and ^***^p < 0.001.

In assessing the RSC, the scale indicated weaker RSC among PIU adolescents than healthy adolescents [median (IQR) for healthy adolescents: 87.0 (74.0 to 99.0); median (IQR) for PIU adolescents: 74.0 (70.0 to 84.0), *p* < 0.001; Figure 2].

**Figure 3:**
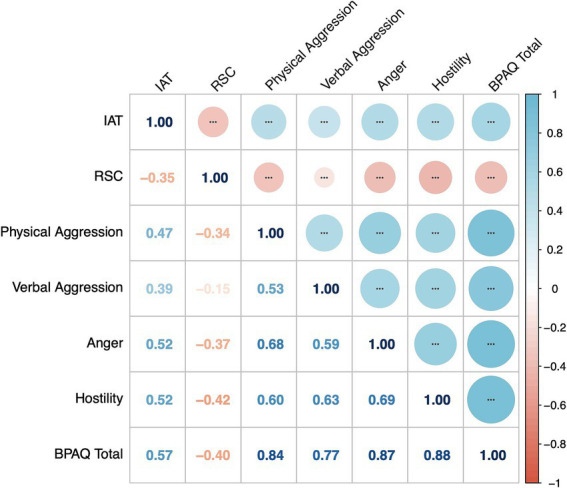
The partial correlation results among the IAT, BPAQ, and RSC The partial correlation scores were presented. IAT, the score of the Internet Addiction Test (IAT-20) which measured the severity of internet use; RSC, reality social connectedness. ^*^p < 0.05, ^**^p < 0.01, and ^***^p < 0.001.

Furthermore, the partial correlation shows a positive correlation between the IAT score and aggression including the total score of BPAQ and four subscales, such as physical aggression, verbal aggression, anger, and hostility (Figure 3).

To explore the quantitative relationship between PIU and aggression, we conducted both univariable regression and multivariable hierarchical regression analyses. The univariable regression showed that the age, gender, residence, and parents’ educational level were significantly correlated with the BPAQ total score (Table 2). Therefore, these variables were included as control factors in the following multivariable analyses.

**Table 2:** Hierarchical regression evaluating RSC as moderator of the relationship between PIU and aggression.

Variable		Univariable	Multivariable, *B*, (95% CI)		
			Model 1	Model 2	Model 3
Age	(12, 19)	0.42 (0.18 to 0.66)^***^		−0.25 (−0.47 to −0.03)^*^	−0.38 (−0.59 to −0.17)^***^
Gender	Boys (*N* = 4,556)				
	Girls (*N* = 4,835)	−1.14 (−1.91 to −0.36)^**^		−1.61 (−2.31 to −0.92)^***^	−1.13 (−1.80 to −0.47)^***^
Only child	No (*N* = 5,170)				
	Yes (*N* = 4,221)	−0.15 (−0.93 to 0.62)		0.28 (−0.46 to 1.02)	0.33 (−0.39 to 1.04)
Residence	City (*N* = 7,416)				
	Town (*N* = 1,262)	−2.15 (−3.28 to −1.03)^***^		−1.44 (−2.49 to −0.40)^**^	−0.89 (−1.89 to 0.10)
	Village (*N* = 713)	7.12 (5.38 to 8.85)^***^		4.72 (3.10 to 6.33)^***^	4.22 (2.69 to 5.75)^***^
Family economy	Average (*N* = 6,967)				
	Quite poor (*N* = 497)	7.47 (3.86 to 11.07) ^***^		4.43 (1.14 to 7.72)^**^	3.08 (−0.04 to 6.21)
	Relatively affluent (*N* = 1,649)	6.28 (4.55 to 8.01) ^***^		3.31 (1.71 to 4.91)^***^	2.05 (0.52 to 3.58)^**^
	Very affluent (*N* = 170)	−2.52 (−3.54 to −1.50) ^***^		−1.68 (−2.62 to −0.74)^***^	−0.83 (−1.73 to 0.06)
	Very poor (*N* = 108)	−4.86 (−7.74 to −1.97) ^***^		−3.02 (−5.63 to −0.41)^*^	−1.59 (−4.08 to 0.90)
Father education level	Primary or below (*N* = 805)				
	Secondary education (*N* = 4,958)	−4.22 (−9.83 to 1.39)^**^		0.37 (−1.00 to 1.74)	0.73 (−0.57 to 2.04)
	Tertiary education (*N* = 3,628)	−3.46 (−4.92 to −2.01)^***^		0.07 (−1.53 to 1.66)	0.64 (−0.88 to 2.16)
Mother education level	Primary or below (*N* = 1,137)				
	Secondary education (*N* = 4,640)	−3.54 (−4.77 to −2.31)^***^		−1.47 (−2.68 to −0.27)^*^	−0.85 (−2.00 to 0.30)
	Tertiary education (*N* = 3,614)	−4.38 (−5.65 to −3.11)^***^		−1.34 (−2.80 to 0.12)	−0.55 (−1.93 to 0.84)
PIU	Health (*N* = 7,336)				
	PIU (*N* = 2,055)		19.42 (18.58 to 20.27) ^***^	19.09 (18.25 to 19.94)^***^	15.47 (14.63 to 16.30)^***^
Reality social connectedness					−0.35 (−0.37 to −0.33)^***^
*p*-value			*p* < 0.001	*p* < 0.001	*p* < 0.001
*R* ^2^			0.177	0.195	0.272
Adj. *R*^2^			0.177	0.193	0.27

^*^p < 0.05, ^**^p < 0.01, and ^***^p < 0.001.

The hierarchical regression showed the impact of individual factors (Table 2). The PIU status was set as the independent variable, whereas the BPAQ total score was set as the dependent variable. In Model 1, PIU explained 17.7% of the variance in the BPAQ total score. Model 2 incorporated basic sociodemographic variables into the analysis, resulting in a modest increase in explanatory power (adjusted *R*^2^ = 0.193, Δ*R*^2^ = 0.016). Model 3 introduced RSC into the linear model, revealing a 1.6% contribution to the BPAQ total score (*R*^2^ = 0.272, adjusted *R*^2^ = 0.270, and Δ*R*^2^ = 0.077). Notably, the RSC showed a negative modulated aggression arising from PIU (*B* = −0.35, 95% CI = −0.37 to −0.32, *p* < 0.001), consistent with the previous partial correlation result.

### 3.3 RSC as a mediator between the PIU and aggression

**Figure 4:**
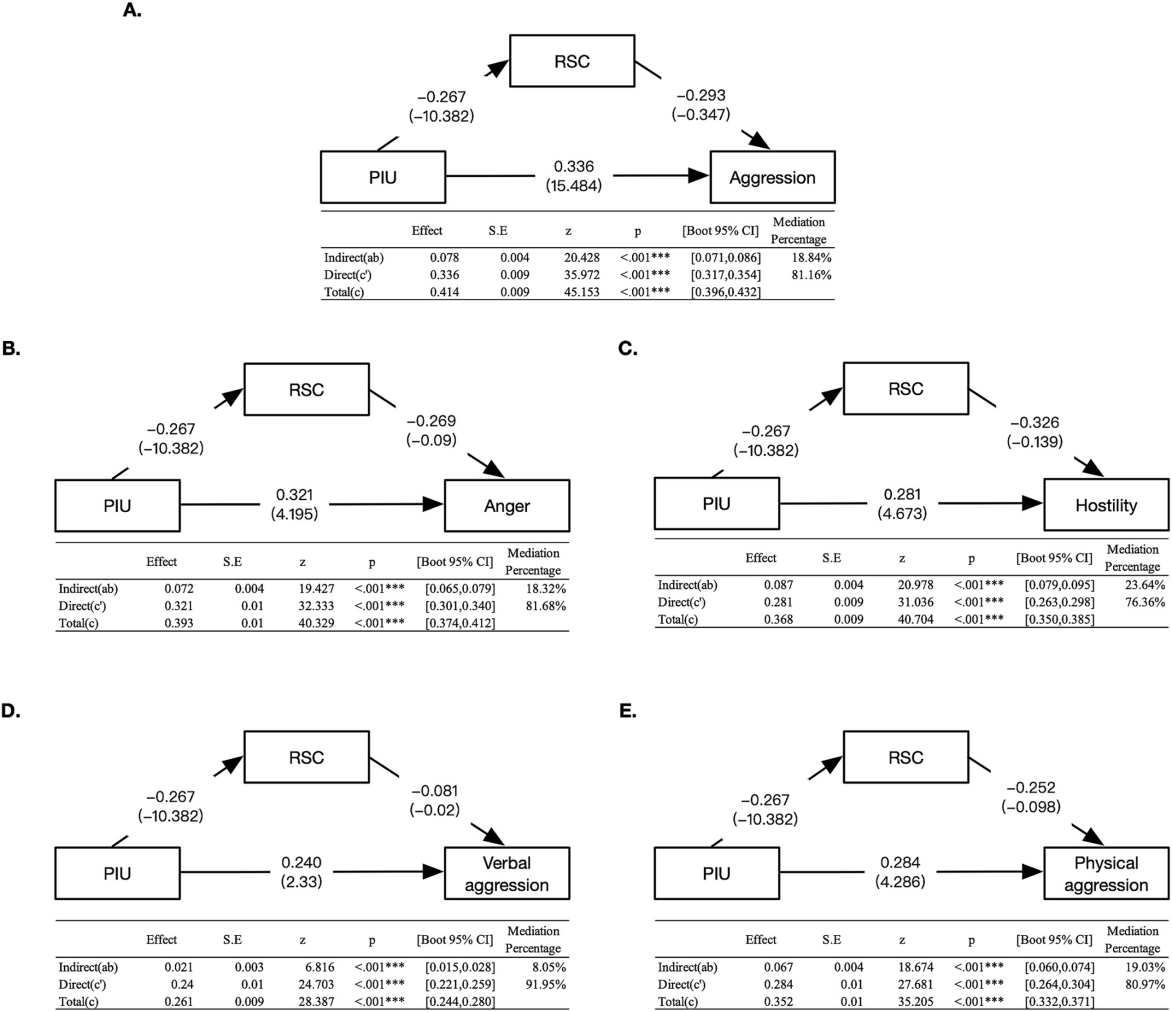
Mediation model with standardized and unstandardized coefficients. The mediation effects of RSC on the relationship between PIU and (**A**. aggression, **B**. Anger, **C**. Hostility, **D**. Verbal aggression, **E**. Physical aggression). Several variables such as age, sex, residence, family economic status was set as covariates in mediation effects analysis. Standardized coefficients (β) are reported, with unstandardized coefficients **(B)** presented in parentheses. All 95% confidence interval [CI] was calculated using 5,000 bootstrap replicates. PIU, problematic Internet use; RSC, reality social connectedness; CI, confidence interval. *p < 0.05, **p < 0.01, and ***p < 0.001.

To explore the potential mediation effect of RSC on the relationship between PIU and aggression, we used the mediation analysis (Figure 4A). We found a significant direct effect [*β* = 0.336, SE = 0.009, 95% CI (0.317, 0.354)] and indirect effect [*β* = 0.078, SE = 0.004, 95% CI (0.071, 0.086)] when RSC was analyzed as a mediation variable. The mediating effect accounted for 18.84% for the total effect.

To examine the specific contributions of aggression subtypes, we used the four subscales of the BPAQ—anger, hostility, verbal aggression, and physical aggression—as independent variables to model the indirect effects of RSC (Figures 4B–E). Consequently, RSC showed the significant indirect effects: anger *β* = 0.072, 95% CI (0.065, 0.079), SE = 0.004; hostility *β* = 0.087, 95% CI (0.079, 0.095), SE = 0.004; verbal aggression *β* = 0.021, 95% CI (0.015, 0.028), SE = 0.003; and physical aggression *β* = 0.067, 95% CI (0.060, 0.074), SE = 0.004. The mediation effect of RSC could explain the highest proportion to hostility (23.64%), followed by the physical aggression (19.03%), anger (18.32%), and verbal aggression (8.05%).

## 4 Discussion

First, our study demonstrated a significant association between PIU and aggression, encompassing the four subtypes: anger, hostility, physical aggression, and verbal aggression. Second, RSC was negatively correlated with both PIU and anger. Finally, mediation analysis revealed a significant mediating effect of RSC in the relationship between PIU and aggression.

Significant correlations were found between IAT, which reflected the severity of PIU, and aggression including the four subtypes. This result is consistent with an Israeli study that found a positive relationship between internet addiction and aggression (4). Similar results have been found in other studies (7, 37, 38). Collectively, these studies suggest that PIU may foster aggression across various cultures and economic situations. The internet world provides a virtual anonymous world that the moral constraints on aggression are also down (39). Teenagers are more likely to be exposed to impulsive and violent content. Additionally, the community system constructed by the internet breaks down traditional geographic isolation, making it easier to form communities based on shared interests. This system facilitates access to such content, creating a positive feedback loop that further reinforces exposure to and engagement with impulsive and violent materials. This association is particularly concerning given our adolescent sample. Compared with other age groups, adolescents are at their developmental peak while facing identity transitions influenced by unique psychological and social factors. Studies have found that physical aggression tends to rise during the sixth and seventh grades (40). Additionally, one study highlights that physical aggression reaches its peak at age 15, whereas social aggression peaks at age 14 (41). Consequently, the confluence of aggression and PIU among adolescents necessitates heightened attention and targeted intervention strategies.

Our findings show that RSC significantly mediates the relationship between PIU and aggression points to the protective role of RSC. Previous studies have shown that RSC can alleviate aggression, which is consistent with other studies (42–44). The basic mechanism of indirect effect (PIU → RSC → aggression) is that, as PIU increased, individuals spend less time on realistic socializing, leading to a reduction in RSC. This decline in RSC would weaken the RSC’s protection against aggression (43). Beyond that, another potential mechanism underlying this relationship involves emotional regulation. PIU is often associated with negative emotions, especially loneliness, anxiety, and depression (45–48). These harmful negative emotions can lead people to withdraw from real-world communication. When combined with those negative factors, the protective role will be weakened.

Among the four aggression subtypes, RSC exhibited a stronger protective effect against hostility, physical aggression, and anger, but a weaker protective effect against verbal aggression. Types of internet use among adolescents may be a key regulator. Generally, social media and online gaming are the main forms of internet use among teenagers. In China, according to government research, mobile phone is the first choice for accessing the internet among minors (49), and wearable devices, such as smartwatches, are the second choice for accessing the internet. Interestingly, even some of the non-traditional network devices with screens that can access the internet (e.g., smart speakers, smart desk lamps, electronic dictionaries, etc.) have also become a method for minors to access the internet. This may be due to strong parental supervision as well as government regulation, resulting in minors being restricted from accessing traditional online devices. However, these devices are often not capable of running online games due to device performance issues and are more likely to use social media. So, the problems associated with social media may be more pronounced in Chinese adolescents. Given the communication style prevalent on social media platforms, verbal aggression may be particularly dominant among adolescents. Moreover, the anonymity of the web lowers the barriers of verbal aggression (50).

## 5 Limitation

This study has several limitations. Although we emphasized that the data would be anonymized for this study and participants had the option to submit anonymously, the involvement of schools and guardians may have influenced participants to provide socially desirable responses. Additionally, there are also confounders in this study that may affect further generalization of the results. For example, the study did not include some psychometric indicators such as loneliness, anxiety, depression, etc. It also lacked measurement such as relationships of family members that might influence participant behavior. Good family dynamics can play a protective role in the development of aggression (51), whereas family conflict may exacerbate its development (52).

Although we selected middle school students from four provinces in China, factors such as geographic variation, differences in economic development, and cultural diversity may not capture the full diversity of the Chinese adolescent population. Additionally, the cross-sectional design of this study precludes the establishment of causal relationships. Future research should employ longitudinal designs to better understand the causal pathways between PIU, RSC, and aggression.

## 6 Conclusion

In conclusion, our findings highlight the aggression associated with PIU, especially among middle school students who are in a special period of mental and physical development. These findings suggest that targeted interventions focusing on enhancing the RSC among adolescents with PIU could potentially reduce aggression. Therefore, school-based activities designed to promote healthy interpersonal relationships, along with social programs aimed at foresting supportive family environments, can serve as effective strategies to prevent aggression in adolescents with PIU. These interventions not only assist in reducing unhealthy online behaviors but also help prevent incidents of adolescent conflict related to internet addiction.

## Data Availability

The data analyzed in this study is subject to the following licenses/restrictions: the data supporting the findings of this study can be obtained upon request from the corresponding author. However, the data are not publicly accessible due to privacy and ethical considerations. Requests to access these datasets should be directed to xujiajun120@126.com.
